# A versatile pretargeting approach for tumour-selective delivery and activation of TNF superfamily members

**DOI:** 10.1038/s41598-017-13530-w

**Published:** 2017-10-16

**Authors:** Yuan He, Peter E. van Bommel, Douwe F. Samplonius, Edwin Bremer, Wijnand Helfrich

**Affiliations:** 1 University of Groningen, University Medical Center Groningen (UMCG), Department of Surgery, Laboratory for Translational Surgical Oncology, Groningen, The Netherlands; 2 University of Groningen, University Medical Center Groningen (UMCG), Department of Experimental Haematology, section Immunohematology, Groningen, The Netherlands; 3 University of Exeter Medical School, St Luke’s Campus, Exeter, Devon, UK

## Abstract

TNFR superfamily (TNFRSF) members have important immunoregulatory functions and are of clear interest for cancer immunotherapy. Various TNFRSF agonists have been clinically evaluated, but have met with limited efficacy and/or toxicity. Recent insights indicate that ‘first-generation’ TNFRSF agonists lack efficacy as they do not effectively cross-link their corresponding receptor. Reversely, ubiquitous TNFRSF receptor(s) cross-linking by CD40 and Fas agonistic antibodies resulted in dose-limiting liver toxicity. To overcome these issues, we developed a novel pretargeting strategy exploiting recombinant fusion proteins in which a soluble form of TRAIL, FasL or CD40L is genetically fused to a high-affinity anti-fluorescein scFv antibody fragment (scFvFITC). Fusion proteins scFvFITC:sTRAIL and scFvFITC:sFasL induced potent target antigen-restricted apoptosis in a panel of cancer lines and in primary patient-derived cancer cells, but only when pretargeted with a relevant FITC-labelled antitumour antibody. In a similar pretargeting setting, fusion protein scFvFITC:sCD40L promoted tumour-directed maturation of immature monocyte-derived dendritic cells (iDCs). This novel tumour-selective pretargeting approach may be used to improve efficacy and/or reduce possible off-target toxicity of TNFSF ligands for cancer immunotherapy.

## Introduction

The TNF-receptor superfamily (TNFRSF) serves various key immunoregulatory functions and includes Death Receptors that trigger apoptosis in cancer cells and receptors that provide co-stimulatory signals to anti-tumour T cells. Accordingly, various agonistic TNFRSF antibodies and recombinant forms of TNFSF ligands have been clinically evaluated^1–6^. For instance, recombinant TRAIL or agonistic TRAIL-receptor antibodies were well-tolerated, but yielded only limited clinical efficacy. Reversely, ubiquitous CD40 or Fas cross-linking by recombinant ligand or agonistic antibodies induced dose-limiting liver toxicity^7,8^ and met with no or only limited clinical benefit^4,9,10^. The disappointing clinical activity of these recombinant soluble TNFSF ligands is attributable to various factors, including short serum half-life, ubiquitous expression of the cognate TNFRSF receptor(s), presence of competing decoy receptors and a reduced capacity to activate some of the cognate TNFRSF. In particular, sTRAIL, sFasL or sCD40L fail to effectively trigger down-stream signalling pathways of TRAIL-R2, Fas and CD40, respectively, as these receptors are only effectively activated by membrane-bound or secondarily multimerized cognate ligands^7,11,12^. In this respect, both sFasL and sCD40L require at least hexamerization in order to induce receptor activation.

Previously, we demonstrated that activity of recombinant homotrimeric TNFSF ligands can be fully restored in a target antigen-restricted manner by their genetic fusion to a cancer cell-directed scFv antibody fragment. This approach has yielded a broad panel of scFv:TNFSF-ligand fusion proteins directed against target antigens overexpressed on solid cancers (e.g. EpCAM, EGFR, MCSP and CD47) or haematological malignancies (e.g. CD7, CD19, CD20, CD33 and CLL-1^13–20^. Unfortunately, essentially all of the currently known and clinically applied target antigens in antibody-based approaches are not exclusively expressed on cancer cells. Indeed, on-target/off-tumour activity and toxicity remain major concerns for all antibody-based therapies, most notably for BiTEs and CAR-T cells^21,22^. Moreover, it is well established that both solid and non-solid malignancies show antigen heterogeneity due to genomic instability, epigenetic alterations and microenvironmental differences^23,24^.

To address these issues, we here report on a two-step approach which involves pretargeting of cancer cells with fluorescein-labelled anticancer antibodies, followed by treatment with a recombinant scFv:TNFSF fusion protein with high-affinity binding capacity for fluorescein derivatives. These scFv:FITC:sTNFSF fusion proteins only gain full agonistic activity upon binding to cancer cells pretargeted with a FITC-labelled antibody. Using this two-step approach, tumour-selective pro-apoptotic activity of fusion proteins scFvFITC:sTRAIL and scFvFITC:sFasL was achieved towards various cell lines and primary patient-derived cancer cell types. In a similar pretargeting setting, fusion protein scFvFITC:sCD40L promoted tumour-directed maturation of immature monocyte-derived dendritic cells (iDCs).

## Results

### Two step pretargeting with scFvFITC:sTRAIL selectively induces apoptosis in leukaemia cells

**Figure 1:**
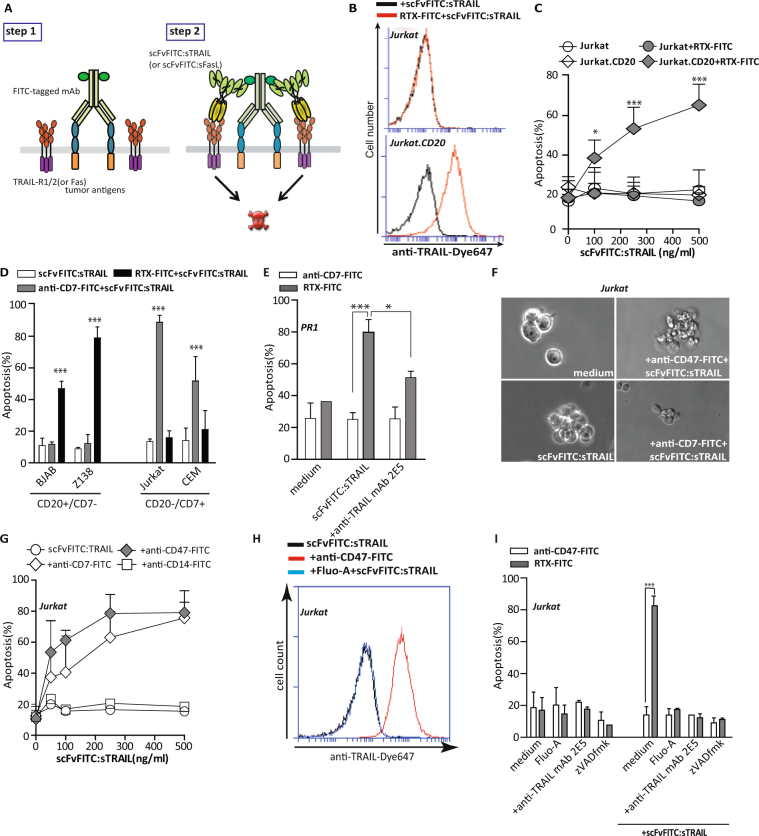
CD20-selective binding and apoptosis induction by scFvFITC:sTRAIL in CD20^pos^ leukaemia cells pretargeted with RTX-FITC (**A**) Schematic representation of scFvFITC:sTRAIL (or scFvFITC:sFasL)-mediated apoptosis in cancer cells pretargeted with FITC-labelled anti-tumour MAbs. (**B**) Flow cytometric analysis of scFvFITC:sTRAIL binding to Jurkat.CD20 cells pretargeted with RTX-FITC and Jurkat cells. (**C**) Jurkat.CD20 and Jurkat cells were pretargeted with RTX-FITC followed by treatment with an increasing dose of scFvFITC:sTRAIL (up to 500 ng/ml) for 24 h. (**D**) A panel of leukaemia cell lines was pretargeted either with anti-CD20-FITC or anti-CD7-FITC, followed by treatment of scFvFITC:sTRAIL (100 ng/ml) for 24 h. (**E**). PR1 cells were incubated with RTX-FITC and followed by treatment of scFvFITC:sTRAIL (500 ng/ml) with or without anti-TRAIL mAb (5 µg/ml). (**F**) Representative light microscopic photos of Jurkat cells upon scFvFITC:sTRAIL treatment. (**G**) Jurkat cells were first incubated with anti-CD14-FITC, anti-CD7-FITC, or anti-CD47-FITC, followed by treatment of an increasing dose of scFvFITC:sTRAIL (up to 500 ng/ml) for 24 h. (**H**) Flow cytometric analysis of scFvFITC:sTRAIL binding to Jurkat cells pretargeted with anti-CD47-FITC with or without non-fluorescent fluorescein. (**I**) CD47-FITC-pretargeted Jurkat cells were treated with scFvFITC:sTRAIL in the presence or absence with anti-TRAIL MAb (5 µg/ml), pan-caspase inhibitor zVADfmk (20 µM), or Fluo-A(6.25 µM) for 24 h. Apoptosis in all experiments was determined by Annexin V/PI staining. All graphs represent mean ± SD. Statistical analysis was performed using two-way ANOVA (*p < 0.05, **p < 0.01, ***p < 0.001, n.s. not significant).

To gain initial proof-of-concept, we used CD20-based pretargeting with FITC-labelled rituximab (RTX) in Jurkat.CD20 and wt CD20^neg^ Jurkat cells. As expected, scFvFITC:sTRAIL only bound to Jurkat.CD20 cells upon pretargeting with RTX-FITC, but not to CD20^neg^ wt Jurkat cells (Fig. 1B). Correspondingly, scFvFITC:sTRAIL dose-dependently induced apoptosis in Jurkat.CD20, but not in Jurkat cells, upon pretargeting with RTX-FITC (Fig. 1C). Similar pretargeting activity by scFvFITC:sTRAIL was detected towards CD20^pos^/CD7^neg^ B-cell lines BJAB, Z138 and PRI only when pretargeted with RTX-FITC, with no activity upon pretargeting with an irrelevant FITC-labelled anti-CD7 antibody (Fig. 1D and E). Induction of apoptosis by scFvFITC:sTRAIL in RTX-FITC pretargeted CD20^pos^ PR1 leukaemia cells was significantly inhibited in the presence of excess amounts of TRAIL-neutralizing mAb 2E5 (Fig. 1E), indicating that apoptotic activity was due to activation of TRAIL-R apoptotic signalling.

Analogously, when CD7^pos^/CD20^neg^ Jurkat or CEM T cell leukemic cells were pretargeted with anti-CD7-FITC, treatment with scFvFITC:sTRAIL dose-dependently induced apoptosis, whereas pretargeting with RTX-FITC did not (Fig. 1D). Similarly, scFvFITC:sTRAIL potently induced apoptosis in CD7^pos^/CD47^pos^/CD20^neg^/CD14^neg^ Jurkat cells when pretargeted with anti-CD7-FITC or anti-CD47-FITC, whereas no apoptosis was induced in the absence of these antibodies or when pretargeted with anti-CD14-FITC (Fig. 1F and G). Importantly, binding as well as pro-apoptotic activity of scFvFITC:sTRAIL was inhibited when treatment was performed in the presence of excess amount of fluorescein analogue Fluo-A (Fig. 1H, and I). Further, apoptotic activity by scFvFITC:sTRAIL in anti-CD47-FITC pretargeted Jurkat cells was blocked in the presence by TRAIL-neutralizing antibody 2E5 or total caspase-inhibitor zVADfmK (Fig. 1I).

### Two step pretargeting with scFvFITC:sTRAIL selectively induces apoptosis in various solid cancer cell types

**Figure 2:**
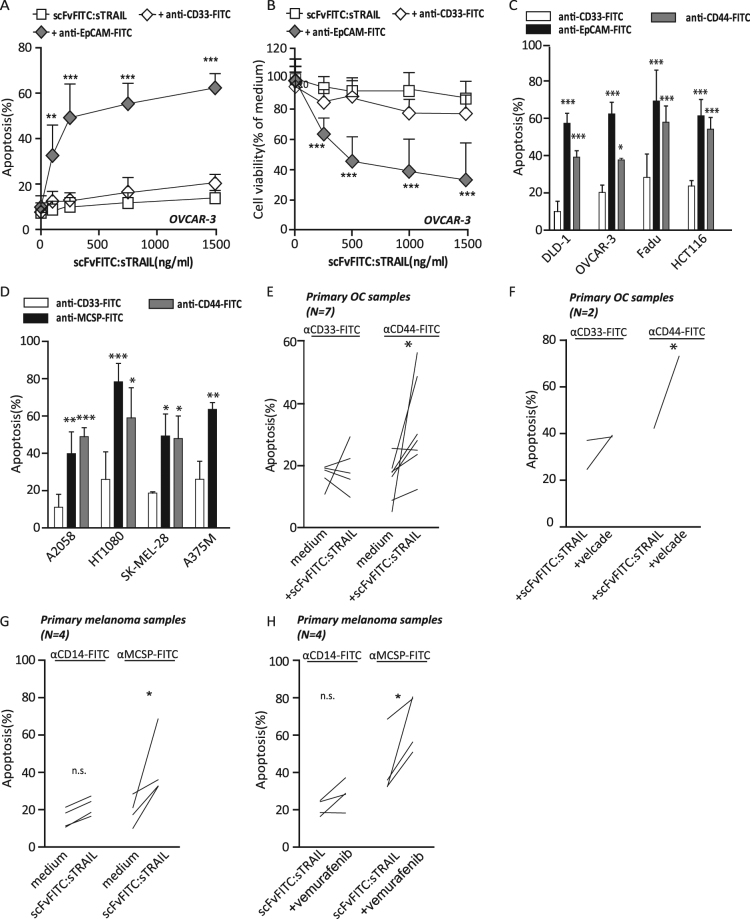
scFvFITC:sTRAIL-mediated apoptosis in solid tumour cells pretargeted with FITC-labelled MAbs. (**A**) OVCAR-3 cells were incubated with anti-CD33-FITC (isotype control) or anti-EpCAM-FITC followed by treatment with an increasing dose of scFvFITC:sTRAIL (up to 1.5 µg/ml) for 24 h. Apoptosis was determined by Annexin V/PI staining. (**B**) In the same experimental setting as (**A**), cell viability of OVCAR-3 cells was determined by MTS assay after 72 h treatment. (**C**) In an extended analysis, a panel of carcinoma cells was pretargeted with anti-CD44-FITC or anti-EpCAM-FITC followed by scFvFITC:sTRAIL treatment (1.5 µg/ml). (**D**) A panel of MCSP^pos^ cancer cells were incubated with anti-MCSP-FITC or anti-CD44-FITC followed by treatment of scFvFITC:sTRAIL (1.5 µg/ml). (**E**) Seven primary patient-derived ovarian samples were pretargeted with anti-CD33-FITC (isotype control) or anti-CD44-FITC followed by treatment of scFvFITC:sTRAIL (1.5 µg/ml). (**F**) Two pretargeted primary OC samples with co-treated with scFvFITC:sTRAIL and Velcade (5 µM) (**G**); Four primary patient-derived melanoma samples were pretargeted with anti-MCSP-FITC followed by treatment of scFvFITC:sTRAIL(1.5 µg/ml). (**H**) Anti-MCSP-FITC-pretargeted primary melanoma samples (N = 4) were co-treated with scFvFITC:sTRAIL and vemurafenib (10 μM). Apoptosis in all experiments was determined by Annexin V/PI staining. All graphs represent mean + SD. Statistical analysis was performed using two-way ANOVA (*p < 0.05, **p < 0.01, ***p < 0.001, n.s. not significant).

To further establish utility of the pre-targeting approach, ovarian carcinoma cell line OvCAR-3 (EpCAM^pos^/CD44^pos^) was pretargeted with anti-EpCAM-FITC or anti-CD44-FITC. Subsequent treatment with scFvFITC:sTRAIL dose-dependently induced apoptosis and reduced cell viability (Fig. 2A–C). In contrast, no apoptosis or loss in cell viability was detected when cells were pretargeted with irrelevant anti-CD33-FITC antibody or upon treatment with anti-EpCAM-FITC or anti-CD44-FITC alone (Fig. 2A–C). Similarly, elimination of MCSP^pos^ cancer cells by scFvFITC:sTRAIL was only observed when pretargeted with anti-MCSP-FITC, whereas pretargeting with an irrelevant anti-CD33-FITC antibody failed to do so (Fig. 2D). Thus, target antigen-selective pro-apoptotic activity of scFvFITC:sTRAIL was detected when cancer cells were pretargeted with a relevant first step FITC-labelled anticancer antibody and is applicable to diverse cancer types.

### Two step pretargeting with scFvFITC:sTRAIL selectively induces apoptosis in patient-derived cancer cells

In a more clinically-relevant setting, short-term cultures of primary patient-derived ovarian cancer (OC) cells were pretargeted with anti-CD44-FITC, upon which treatment with scFvFITC:sTRAIL induced apoptosis in 6 out of 7 treated samples (Fig. 2E). In contrast, pretargeting with control antibody anti-CD33-FITC did not induce apoptosis in 4 out of 5 samples (Fig. 2E). Similar results were obtained for treatment of patient-derived primary melanoma cells with scFvFITC:sTRAIL after pretargeting with anti-MCSP-FITC, with significant induction of apoptosis in 4 out of 4 samples tested (Fig. 2G). Of note, combination of the FITC-pretargeting scFvFITC:sTRAIL strategy with proteasome inhibitor bortezomib significantly enhanced apoptosis in 2 out of 3 primary OC samples when pretargeted with anti-CD44-FITC (Fig. 2F), whereas cotreatment with vemurafenib enhanced scFvFITC:sTRAIL-induced apoptosis in 4 out of 4 patient-derived primary melanoma cell cultures (Fig. 2H). Together, these results demonstrate that scFvFITC:sTRAIL selectively binds to FITC-tagged cells and can subsequently induce apoptosis in multiple types of cancer cells only when pretargeted with appropriate FITC-labelled anticancer antibody.

### Two step pretargeting with scFvFITC:sFasL selectively induces apoptosis in leukaemia cells

**Figure 3:**
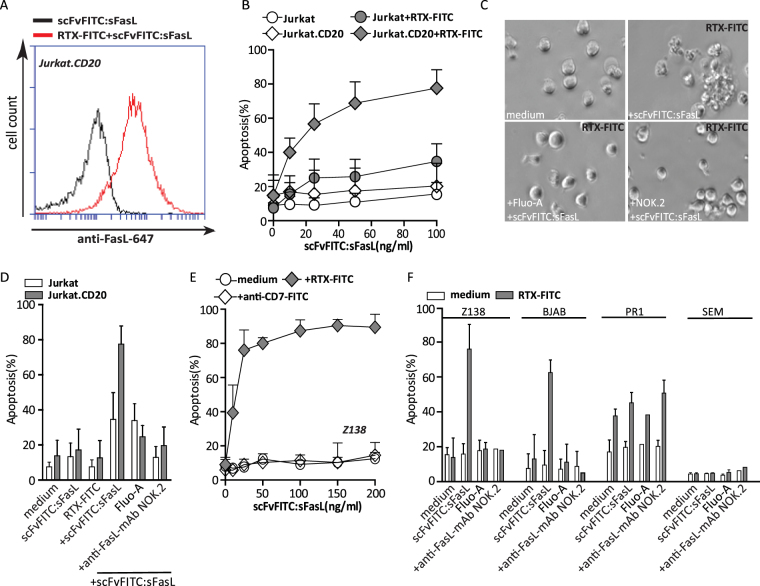
Selective apoptosis by scFvFITC:sFasL in RTX-FITC-pretargeted CD20^pos^ leukaemia cells. (**A**) Flow cytometric analysis of scFvFITC:sFasL binding to Jurkat.CD20 pretargeted with RTX-FITC. (**B**) Jurkat.CD20 and Jurkat cells were pretargeted with RTX-FITC followed by treatment of an increasing dose of scFvFITC:sFasL (100 ng/ml) for 24 h. (**C**) Representative light microscopic photos of Jurkat and Jurkat.CD20 cells upon scFvFITC:sFasL treatment. Jurkat and Jurkat.CD20 cells were pretargeted with RTX-FITC followed by treatment of scFvFITC:sFasL (100 ng/ml) for 24 h in the presence or absence of Fluo-A or anti-FasL MAb(5 μg/ml). (**D**) Z138 cells were pretargeted with anti-CD7-FITC or RTX-FITC followed by treatment of an increasing dose of scFvFITC:sFasL for 24 h. (**E**) CD20-positive leukaemia cells (BJAB, Z138 and PR1) and CD20-negative cells (SEM) were pretargeted with RTX-FITC or anti-CD7-FITC followed by scFvFITC:FasL treatment (100 ng/ml) in the presence or absence of Fluo-A or anti-FasL MAb. Apoptosis in all experiments was determined by Annexin V/PI staining. All graphs represent mean ± SD. Statistical analysis was performed using two-way ANOVA (*p < 0.05, **p < 0.01, ***p < 0.001, n.s. not significant).

Similar to scFvFITC:sTRAIL, scFvFITC:sFasL selectively bound to Jurkat.CD20 only when pretargeted with RTX-FITC (Fig. 3A), whereas limited binding was detected on parental Jurkat cells (Supplementary data 1C). The binding of scFvFITC:sFasL was abrogated when treatment was performed in the presence of excess molar amounts of Fluo-A (Supplementary data Fig. 1D). In line with this, scFvFITC:sFasL dose-dependently induced apoptosis in Jurkat.CD20 cells when pretargeted with RTX-FITC, whereas Jurkat cells remained largely unaffected (Fig. 3B). This pro-apoptotic activity of scFvFITC:sFasL was abrogated in the presence of excess amounts of Fluo-A or FasL-neutralizing mAb NOK.2 (Fig. 3C and D). Further, treatment of either Jurkat.CD20 cells or Jurkat cells with scFvFITC:sFasL alone did not induce apoptosis (Fig. 3C and D). Treatment with scFvFITC:sFasL dose-dependently induced apoptosis in CD20^pos^ Z138 B cell non-Hodgkin’s lymphoma cells pretargeted with RTX-FITC (Fig. 3D) with an EC50 value of 14.9 ng/ml. However, treatment with maximum concentration of scFvFITC:sFasL (200 ng/ml) did not induce apoptosis in non-pretargeted Z138 cells. No apoptotic activity was detected towards Z138 when cells were treated with scFvFITC:sFasL after pretargeting with mock antibody anti-CD7-FITC (Fig. 3E). This pro-apoptotic activity of scFvFITC:sFasL was blocked in the presence of excess amounts of Fluo-A or Fas-neutralizing antibody NOK.2 in Z138 and BJAB cells, although cell death in PR1 cells was not inhibited by the latter (Fig. 3F), which may be due to CD20 cross-linking induced apoptosis, to which PR1 cells are sensitive^16^.

### Two step pretargeting with scFvFITC:sFasL selectively induces apoptosis in solid tumour cells

**Figure 4:**
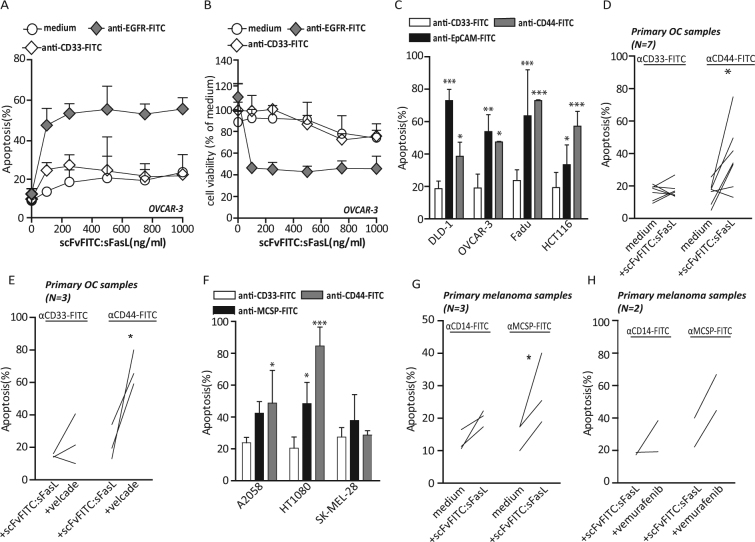
scFvFITC:sFasL-mediated apoptosis in various solid tumour cancer cells pretargeted with FITC-labelled MAbs. (**A**) OVCAR-3 cells were pretargeted either with anti-CD33-FITC or anti-EGFR-FITC followed by treatment of an increasing dose of scFvFITC:sFasL for 24 h. (**B**) OVCAR-3 cells were pretargeted either with anti-CD33-FITC or anti-EGFR-FITC, followed by treatment of an increasing dose of scFvFITC:sFasL for 72 h. (**C**) In an extended analysis, a panel of carcinoma cells were pretargeted with anti-EpCAM-FITC, anti-CD44-FITC and anti-CD33-FITC (isotype control) followed by scFvFITC:sFasL treatment (1 µg/ml). (**D**) Seven primary patient-derived ovarian samples were pretargeted with anti-CD44-FITC or anti-CD33-FITC (isotype control), followed by treatment of scFvFITC:sFasL (1 µg/ml). (**E**) Three pretargeted or non-targeted primary OC samples with co-treated with scFvFITC:sFasL and Velcade (5 nM). (**F**) A small panel of MCSP^pos^ cancer cells were pretargeted with anti-MCSP-FITC anti-CD44-FITC and anti-CD33-FITC (isotype control) followed by treatment of scFvFITC:sTRAIL (1.5 µg/ml). (**G**) Primary patient-derived melanoma samples (N = 3) were pretargeted by anti-MCSP-FITC or anti-CD14-FITC (isotype control) followed by treatment of scFvFITC:sTRAIL. (**H**) Two anti-MCSP-FITC-pretargeted primary melanoma sample were co-treated with scFvFITC:sFasL and vemurafenib (10 μM). Apoptosis in all experiments was determined by Annexin V/PI staining and cell viability was determined by MTS assay. All graphs represent mean ± SD. Statistical analysis was performed using two-way ANOVA (*p < 0.05, **p < 0.01, ***p < 0.001, n.s. not significant).

Treatment of OvCAR3 ovarian cancer cells pretargeted with anti-EGFR-FITC with scFvFITC:sFasL resulted in dose-dependent induction of apoptosis with a corresponding loss in cancer cell viability (Fig. 4A and B). In this experiment, the EC50 value of scFvFITC:sFasL in OVCAR3 cells was calculated to be as low as 100 ng/ml, whereas no apoptosis was induced in OVCAR3 cells upon even when treatment was at the maximum dose of scFvFITC:sFasL. Tumour-selective pro-apoptotic activity of scFvFITC:sFasL was confirmed in a panel of 4 carcinoma cell lines pretargeted with either anti-EpCAM-FITC or anti-CD44-FITC (Fig. 4C). Importantly, analogous treatment with scFvFITC:sFasL also markedly triggered apoptosis in 6 out of 7 primary patient-derived ovarian cancer samples when pretargeted with anti-CD44-FITC, but not with anti-CD33-FITC (4 out of 5) (Fig. 4D). Similarly, in a panel of MCSP-expressing cell lines expressing both MCSP and CD44, treatment with scFvFITC:sFasL induced apoptosis when cells were pretargeted with anti-MCSP-FITC and anti-CD44-FITC, respectively (Fig. 4F). Of note, treatment with scFvFITC:sFasL induced significant apoptosis in 3 out of 3 primary patient-derived melanoma cells pretargeted with anti-MCSP-FITC, whereas no significant increase in apoptosis was observed in cells pretargeted with anti-CD14-FITC (Fig. 4G). Combining scFvFITC:sFasL with bortezomib significantly enhanced apoptosis in 3 out 3 primary ovarian cancer samples when pretargeted with anti-CD44-FITC (Fig. 4E). Similarly, co-treatment with B-RAF inhibitor vemurafenib synergistically triggered cell death in 2 out of 2 primary patient-derived melanoma cell samples (Fig. 4H).

### Two step pretargeting with scFvFITC:sCD40L allows for tumour-localized iDC maturation

**Figure 5:**
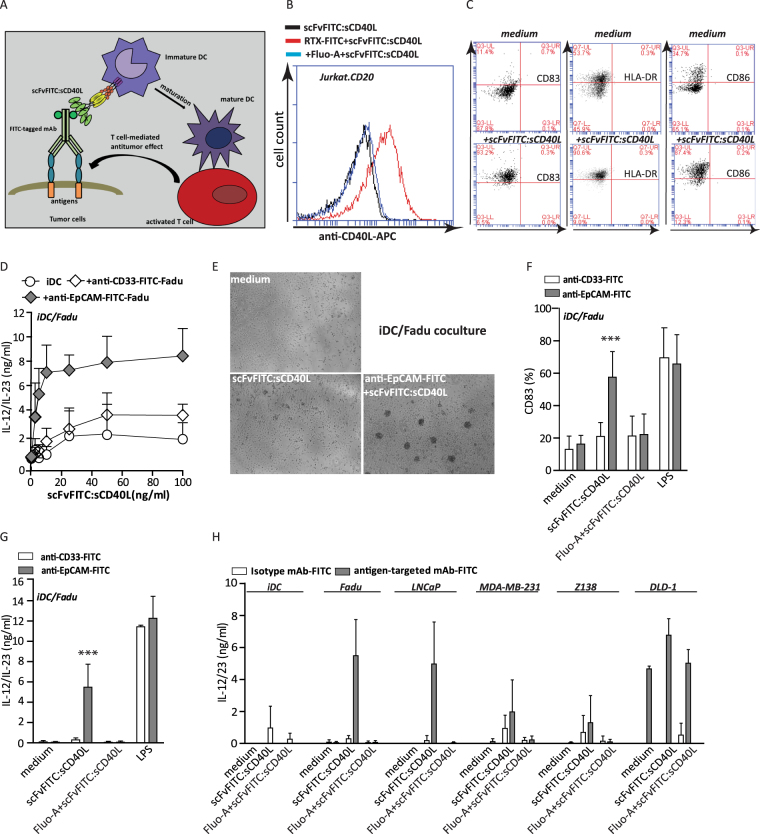
Pretargeting approach for tumour-localized DC maturation using scFvFITC:sCD40L (**A**) Schematic representation of scFvFITC:sCD40L-mediated DC maturation in the presence of cancer cells pretargeted with FITC-labelled anti-tumour MAbs. (**B**) iDC were treated with fresh medium or medium contained scFvFITC:sCD40L (0.5 µg/ml) for 2 days. Subsequently, iDC were harvested and stained with anti-CD83-PE, anti-CD86-FITC, anti-HLA-DR-PE and appropriate isotype controls for assessment of DC maturation. (**C**) Flow cytometric analysis of scFvFITC:sCD40L binding to RTX-FITC-pretargeted Jurkat.CD20 cells in which binding was abrogated by addition of molar excess amounts of Fluo-A. (**D**) iDC were co-cultured with an increasing dose of scFvFITC:sCD40L in the presence or absence of pretargeted or non-targeted FaDu cells for 24 h. DC maturation was assessed by determining IL-12/23 levels in the culture supernatant. **(E)** Representative light microscopic photos of iDC clusters in co-culture with FaDu cells pretargeted with anti-EpCAM-FITC upon scFvFITC:sTRAIL treatment. **(F)** and **(G)** iDC were co-cultured with anti-EpCAM-FITC-pretargeted FaDu or anti-CD33-FITC-pretargeted FaDu cells in the presence or absence of scFvFITC:sCD40L, non- FITC fluorescein or LPS for 24 h. DC maturation was assessed by determining cellular surface CD83 expression and IL-12/23 level in the culture supernatant. (**H)** iDCs were co-cultured with the indicated pretargeted tumour cell lines in the presence or absence of scFvFITC:sCD40L with or without blocking reagent non- FITC fluorescein. iDC maturation was assessed by determination IL-12/23 levels in the culture supernatant. Apoptosis in all experiments was determined by Annexin V/PI staining. Statistical analysis was performed using two-way ANOVA (*p < 0.05, **p < 0.01, ***p < 0.001, n.s. not significant).

Of note, it was previously reported that only when applied at high concentrations sCD40L has capacity to induce iDC maturation (28). Indeed, treatment of iDC with 0.5 µg/ml scFvFITC:sCD40L markedly up-regulated expression levels of CD83, CD86 and HLA-DR (Fig. 5C) which was comparable to up-regulation of these markers upon treatment with LPS (5 µg/ml) (Supplementary data Fig. 2D). First, two step pretargeting of scFvFITC:sCD40L was confirmed using Jurkat.CD20 cells treated with RTX-FITC which resulted in strongly increased fluorescence intensity detected upon secondary anti-CD40L-Dye647 staining (Fig. 5B).

Next, the two step pretargeting approach was evaluated for its capacity to promote tumour-localized iDC maturation using fusion protein scFvFITC:sCD40L (for a schematic representation see Fig. 5A). To this end, FaDu cells (EpCAM^pos^/CD33^neg^), pretargeted with anti-EpCAM-FITC were treated with increasing dose of scFvFITC:sCD40L in the presence of iDCs. This treatment scheme indeed led to a dose-dependent induction of IL-12 production by iDCs, whereas no significant increase IL-12 expression levels were detected when pretargeting with anti-EpCAM-FITC was omitted or when pretargeting was with an irrelevant anti-CD33-FITC antibody (Fig. 5D). Of note, upon treatment with scFvFITC:sCD40L prominent clustering of DCs was observed in co-cultures iDCs and FaDu cells, but only when the latter were pretargeted with anti-EpCAM-FITC. No clustering of DCs was observed when in this treatment scheme FaDu cells were not pretargeted with an irrelevant anti-EpCAM-FITC antibody (Fig. 5E). Concordantly, upon treatment with scFvFITC:sCD40L, CD83 levels on DCs were increased in co-cultures iDCs and FaDu cells, but only when the latter were pretargeted with anti-EpCAM-FITC. No increase in CD83 and IL-12 level was observed when in this treatment scheme FaDu cells were pretargeted with an irrelevant anti-CD33-FITC antibody or when treatment was in the presence of excess amounts of Fluo-A (Fig. 5F and G). Furthermore, analogous scFvFITC:sCD40L treatment schemes in which iDCs were co-cultured with a panel of various cancer cells types significant levels of IL-12 were induced, but only when these cancer types were pretargeted with a FITC-labelled antibody of appropriate specificity (Fig. 5H and Supplementary Table 1). Collectively, these data indicated that scFvFITC:sCD40L promoted antigen-restricted DC maturation in the presence of cancer cells that were pretargeted with a relevant FITC-labelled anticancer antibody preparation.

### No off-target toxicity of scFvFITC:sTRAIL, scFvFITC:sFasL and scFvFITC:sCD40L towards human hepatocytes

Hepatocytes are known to be highly sensitive to toxicity induced by cross-linked and/or aggregated forms of FasL and CD40 agonists. To access for off-target toxicity, IHH hepatocytes (CD44^pos^) were treated with scFvFITC:FasL or scFvFITC:sCD40L. This treatment showed no signs toxicity towards IHH cells. In contrast, apoptosis induction in IHH cells was observed when these were pretargeted with anti-CD44-FITC (Supplementary Fig. 2A).

Next, we wondered whether leukaemia cells displaying scFvFITC:sFasL after appropriate two step pretargeting would induce off-target bystander toxicity in hepatocytes while passing through the liver. To mimic this *in vitro*, IHH cells were cultured in the presence of Ramos B cells pretargeted (or not) with anti-CD19-FITC or the irrelevant antibody anti-CD7-FITC. Subsequent treatment with scFvFITC:sFasL did not induce apoptosis in IHH cells in any of the treatment conditions assessed (Supplementary Fig. 2B and 2C). These data suggest that even in the continued presence of leukaemia cells displaying a surplus scFvFITC:sFasL molecules, only minimal innocent bystander toxicity is induced in hepatocytes.

## Discussion

Here we described a novel two step approach that pretargets cancer cells with a FITC-labelled antibody directed at a tumour-associated cell surface antigen followed by treatment with an essentially inactive scFvFITC:TNFSF fusion protein. As a result of its selective immobilization at the tumour cell surface the inactive scFvFITC:TNFSF fusion protein locally regains its full agonistic activity towards its cognate TNFRSF receptor.

Our data confirm that fusion proteins scFvFITC:sTRAIL and scFvFITC:sFasL, indeed regain potent tumour-selective pro-apoptotic activity towards various types of cancer, both cancer lines and freshly isolated patient-derived primary cancer cells, but only when pretargeted with a relevant FITC-labelled anti-tumour antibody. Similarly, the capacity of fusion protein scFvFITC:sCD40L to promote tumour-directed maturation of immature monocyte-derived dendritic cells (iDCs) was significantly enhanced when tumour cells were pretreated with a relevant FITC-labelled anti-tumour antibody.

Toxicity of TNFSF ligands like FasL and CD40L towards hepatocytes has been a major hurdle for their clinical application. Our results indicated that hepatocytes (IHH cells) were resistant to prolonged treatment with relative high concentrations of scFvFITC:sTRAIL (up to 1000 ng/ml), scFvFITC:sFasL (up to 500 ng/ml) or scFvFITC:sCD40L (up to 500 ng/ml) (Supplementary data Fig. 2A). However, significant apoptosis was induced by scFvFITC:sFasL in CD44^pos^ IHH cells when pretargeted with an anti-CD44-FITC mAb. This is in line with high sensitivity of hepatocytes to Fas cross-linking agonists. This indicated that also for this pretargeting approach suitability of potential target antigens must be carefully evaluated both *in vitro* and *in vivo* to assess possible toxicity towards normal cell types expressing the same target antigen. In this respect, it is of particular interest that the activity of our TNFSF pretargeting approach can be rapidly attenuated by the application of fluorescein in the event of adverse effects. Sodium fluorescein is an FDA-approved fluorescent imaging agent (e.g. Fluorescite) for diagnostic retina and iris angiography^25^. Intravenous administration of 500 mg sodium fluorescein is well tolerated, has fast pharmacokinetics and shows excellent distribution into the interstitial space after.^25^. In a previous study, the activity of anti-FITC chimeric antigen receptor (CAR) T cells was shown to be effectively controlled and attenuated by fluorescein^26,27^.

Obviously, the efficacy of our pretargeting approach is dependent on the expression level of the target antigen of choice. For example, on primary melanoma cells expression of CD44 is higher than MCSP, which translated into a higher efficacy of the scFvFITC:sTRAIL when pretargeted with the anti-CD44 FITC-labelled antibody (Supplementary Fig. 1B and E). Thus, highly over-expressed tumour-associated antigens appear most suited for FITC-based pre-targeting.

It is well established that intratumoural heterogeneity may severely hamper the efficacy of antibody-based therapies directed at one specific target antigen^24^. The FITC-based pretargeting strategy may be used to circumvent this problem by combining two or more different FITC-labelled anticancer antibodies. Studies evaluating the various possible benefits of our pretargeting approach to simultaneously target multiple antigens or different TNFSF ligands to tumour cells, endothelial cells and/or immune cells in the tumour microenvironment are currently on-going.

This pre-targeting approach may be applied to various other TNFSF family members, particularly those that are known to require oligomerization for effective induction of cognate receptor signalling. In this respect, the 4-1BB/4-1BBL signalling axis may be of particular interest as it is crucial in providing co-stimulation to tumour-reactive TILs. Previously, 4-1BBL was shown to require higher order oligomerization to activate 4-1BB signalling^28^. However, ubiquitous 4-1BB activation by agonistic agents is associated with severe liver toxicity in humans^29^. Therefore, 4-1BBL appears of particular interest to be used as TNSF ligand in our pretargeting approach and as such it is currently being explored.

Taken together, in this study we provide proof-of-concept for pretargeting of both pro-apoptotic and co-stimulatory TNFSF family members using readily available FITC-labelled anticancer antibodies. This two-step pretargeting approach may be of use to improve efficacy and/or attenuate possible off-target toxicity of TNFSF ligands for cancer immunotherapy.

## Material and Methods

### Antibodies and reagents

FITC-labelled anti-CD7, anti-CD14, anti-CD19, anti-CD20 and anti-CD47 antibodies (Immunotools, Germany). Anti-EGFR-FITC, anti-MCSP-FITC and anti-TRAIL-PE (Santa Cruz Biotechnology, Germany). Anti-EpCAM-FITC (Stemcell, France), anti-CD44-FITC and anti-CD40L-APC (eBioscience, USA). Trastuzumab-FITC and rituximab-FITC (RTX-FITC) were prepared using commercial FITC conjugation kits (Sigma Aldrich, USA); anti-FasL-PE (Ancell, Bayport, USA) anti-TRAIL-Dye647 (ENZO Life Sciences, USA); TRAIL ELISA kit (Abcam, UK). 5-aminofluorescein (abbreviated as Fluo-A) (Sigma-Aldrich, USA,). BrafV600E inhibitor vemurafenib and proteasome inhibitor bortozomib (Millennium pharmaceuticals, MA) were dissolved at 10 mM in DMSO; IL-12/23 kit (R&D system, USA). Fugene-6 transfection reagent (Promega, Netherlands). IRDye700DX NHS-ester coupling kit (LI-COR, USA).

### Cell lines and primary patient-derived cancer cells

Jurkat, SEM, CEM, BJAB, Z138, HT1080, FaDu, A375m, DLD-1 and OVCAR-3 cell lines were purchased from the ATCC (Rockville, MD). Jurkat.CD20 cells were generated by transfection with pCMV-CD20. Cells lines were authenticated by Short Tandem Repeat analysis and routinely screened for mycoplasma. Immortalized human hepatocytes were from Department of Pediatrics, UMCG. Tumour samples from cancer patients were collected at surgery after informed consent. Cells were cultured in RPMI 1640 or DMEM (10% FCS, 37 °C, 5% CO2). Primary melanoma cells were analysed for CD44 and MCSP expression (Supplementary data Fig. 1B and C). The study protocols were approved by the Institutional Ethics Committees of University Medical Centre Groningen (METC 2011.206 and METC 2012/330 respectively) and informed consents were obtained from all donors. All methods were performed in accordance with the relevant guidelines and regulations.

### Isolation, preparation and phenotyping of DCs

Peripheral blood mononuclear cells (PBMCs) were obtained from healthy volunteers after informed consent using density gradient centrifugation. Monocytes were isolated from PBMCs using anti-CD14-coated magnetic beads (Miltenyi Biotec, USA). For generation of immature DCs (iDCs), monocytes (3 × 10^6^/ml) were cultured in the presence of 500 U/ml GM-CSF and 1000 U/ml IL-4 for 7 d. As positive control, iDCs were treated with 1 μg/ml LPS for 48 h. DC phenotype was flow cytometrically analysed for CD83, CD14, HLA-DR and CD86 expression.

### Construction scFvFITC:TNFSF fusion proteins

DNA encoding a high-affinity anti- fluorescein scFv^26^ was inserted into SfiI and NotI restriction sites of plasmids pEE14scFv:sTRAIL, pEE14scFv:sFasL and pEE14scFv:sCD40L, yielding pEE14-scFvFITC:sTRAIL, pEE14-scFvFITC:sFasL and pEE14-scFvFITC:sCD40L, respectively. Fusion protein scFvFITC:sTRAIL, scFvFITC:sFasL and scFvFITC:sCD40L were produced using CHO-K1 cells^30^.

### Assessment apoptosis and cell viability

Phosphatidylserine (PS) exposure was analysed using Annexin-V-APC. Cell viability was assessed by MTS assay (Promega Benelux, Netherlands). Each experimental and control group was performed in triplicate.

### Binding scFvFITC:TNFSF to cancer cells pretargeted with RTX-FITC

Jurkat and Jurkat.CD20 cells were incubated with RTX-FITC at 4 °C for 1 h, followed by two washes with PBS, and then incubated with scFvFITC:TNFSF, Binding was evaluated by flow cytometry using an APC-labelled antibody directed against the respective TNF-ligand at 4 °C for 45 min.

### Apoptosis induction

Leukaemia cells (5 × 10^4^/well) and solid cancer cells (3 × 10^4^/well) were seeded in a 48-well plate and pretargeted with the indicated FITC-labelled anti-tumour antibody. Unbound antibody was removed by washing and followed by treatment with medium only, scFvFITC:sTRAIL or scFvFITC:sFasL. Synergistic effects of scFvFITC:sTRAIL with velcade (5 μM) or vemurafenib (10 µM) were evaluated using primary patient-derived cancer cells after pretargeting with the indicated FITC-labelled antibody and treatment with scFvFITC:sTRAIL. After 24 h, apoptosis induction was evaluated as indicated.

### Tumour-localized DC maturation by scFvFITC:sCD40L

Tumour-localized maturation of iDC’s by scFvFITC:sCD40L was evaluated by pretargeting (or not) cancer cells with the indicated FITC-labelled anticancer antibodies. In short, iDCs (Effector cells) were mixed with cancer (Target) cells at an E:T cell ratio of 5:1 and then treated with scFvFITC:sCD40L. In a control experiment, competing agent Fluo-A was added. After 48 h, DC activation was evaluated by CD83 expression using flow cytometry and IL-12 secretion by ELISA.

### Assessment off-target toxicity

Off-target toxicity of the scFvFITC:TNFSF pretargeting approach towards hepatocytes was assessed using CD44^pos^ immortal human hepatocytes (IHH)^31^ subjected *in vitro* to scFvFITC:TNFSF alone or in the presence of Ramos cells pretargeted with anti-CD19-FITC. After 24 h apoptosis induction in IHH cells was separately evaluated.

### Statistical analysis

Statistical analysis was performed by one-way ANOVA followed by Tukey-Kramer post-test or, where indicated, by two-sided Student’s t-test using Prism software. P < 0.05 was defined as a statistically significant difference. Where indicated *P < 0.05; **P < 0.01; ***P < 0.001.

## Electronic supplementary material

supplementary information
