# Supplementary material for: A versatile pretargeting approach for tumour-selective delivery and activation of TNF superfamily members
Source: Sci Rep. 2017 Oct 16;7:13301. doi: 10.1038/s41598-017-13530-w (PMC5643434; doi:10.1038/s41598-017-13530-w)
Supplement: Supplementary file 1 — supplementary information [file 41598_2017_13530_MOESM1_ESM.pdf]

A versatile pretargeting approach for tumour-selective delivery and activation of TNF superfamily members

Yuan He[1], Peter E. van Bommel[1], Douwe F. Samplonius[1], Edwin Bremer[2,3#] and Wijnand Helfrich[1#*]

Author list	address
Yuan he	[1]University of Groningen, University Medical Center Groningen (UMCG), Department of Surgery, Laboratory for Translational Surgical Oncology, Groningen, The Netherlands;
Peter E. van Bommel	
Douwe F. Samplonius	
Edwin Bremer	University of Groningen, University Medical Center Groningen (UMCG), Department of Experimental Haematology, section Immunohematology, Groningen, The Netherlands. [3]University of Exeter Medical School, St Luke's Campus, Exeter, Devon, UK
Wijnand Helfrich	University of Groningen, University Medical Center Groningen (UMCG), Department of Surgery, Laboratory for Translational Surgical Oncology, Groningen, The Netherlands;

[#] contributed equally; *corresponding author

Key words: cancer immunotherapy; pretargeting, apoptosis, TNFR superfamily

The authors declare no potential conflicts of interest.

supplementary Fig. 1

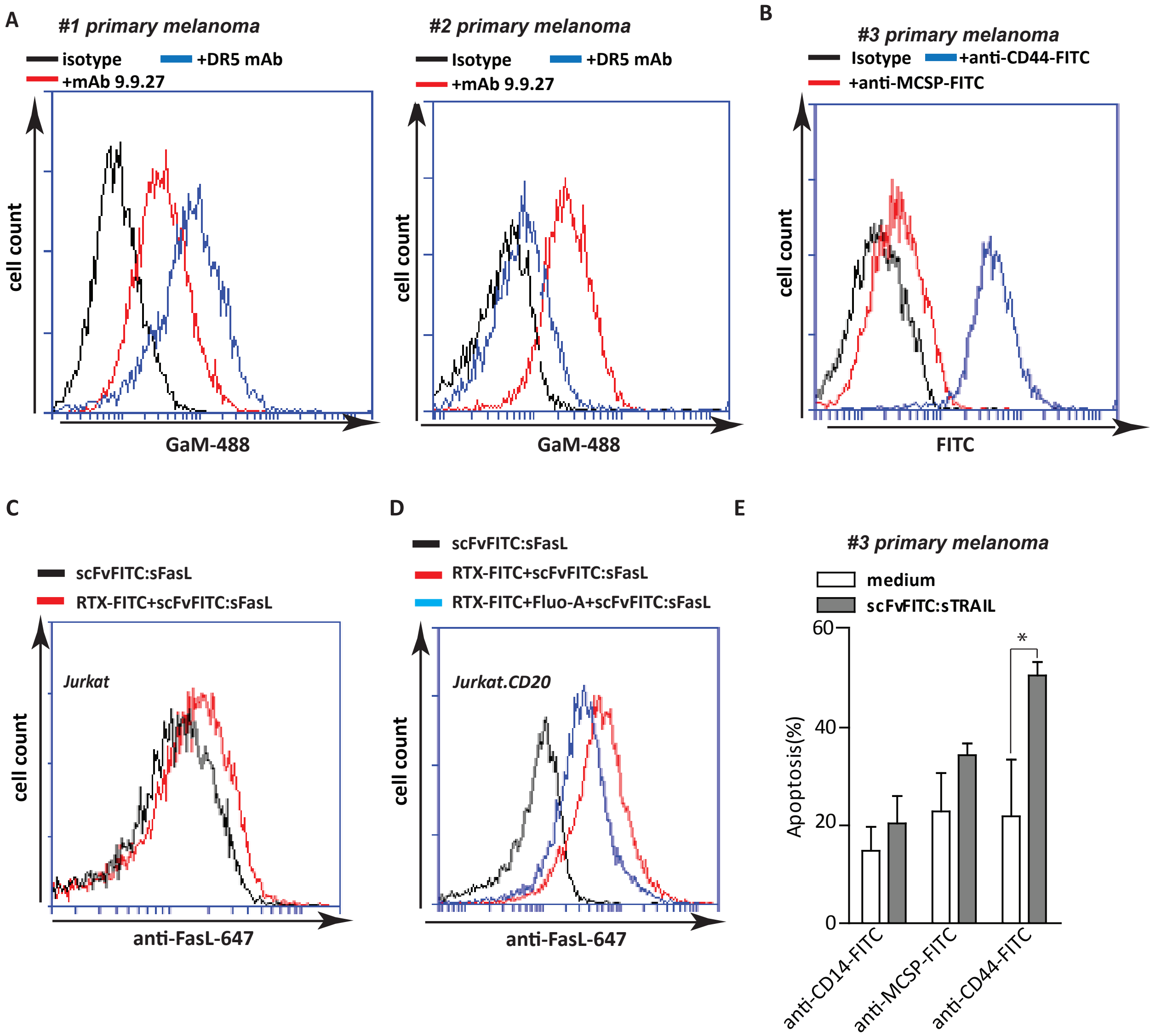

Supplementary Fig.1 (A) MCSP and TRAILR2 expression were determined in primary patient-derived melanoma sample NO.1 and NO.2. MCSP and TRAILR2 expression were determined in primary patient-derived melanoma sample 2. (B) CD44 and MCSP expression were determined in primary melanoma sample NO.3 (C) Primary patient-derived melanoma cells were incubated with anti-MCSP-FITC or anti-CD440FITC, followed by treatment of scFvFITC:sTRAIL (D)Flow cytometric analysis of scFvFITC:sFasL binding to Jurkat cells. (E) Binding of scFvFITC:sFasL to RTX-FITC-labelled Jurkat.CD20 that blocked by addition of FITC was determined by flow cytometry analysis (n=3).

Apoptosis was assessed by Annexin V/PI. Statistical analysis was performed using two-way ANOVA (* $p < 0.05$, ** $p < 0.01$, *** $p < 0.001$, n.s. not significant)

Supplementary Fig.S2

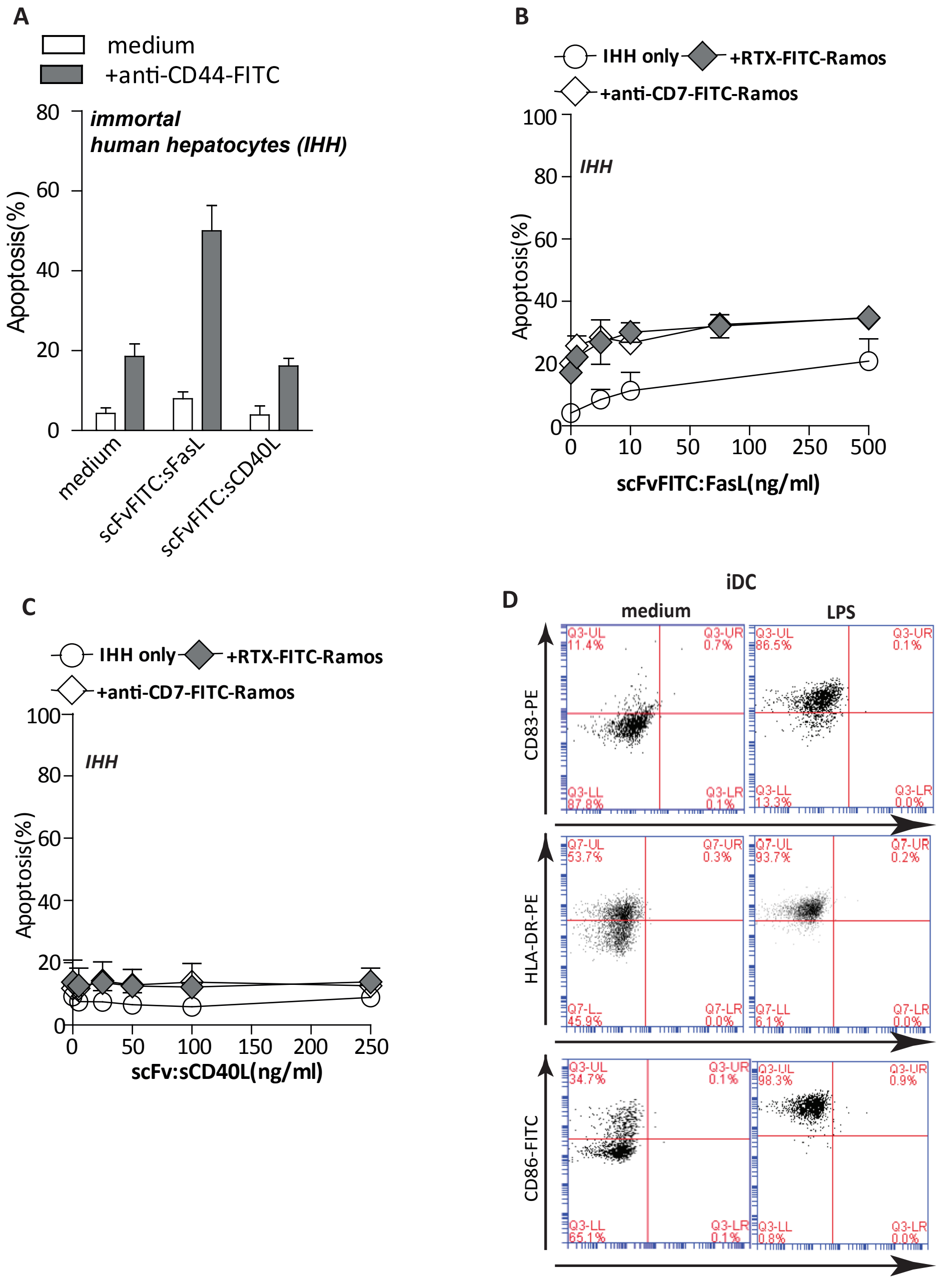

Supplementary Fig.2 (A) IHH were first labeled with or without anti-CD44-FITC and subsequently treated either with scFvFITC:sFasL (100 ng/ml) or scFvFITC:sCD40L (100 ng/ml). (B) IHH were treated with an increasing dose of scFvFITC:sFasL (max. 500 ng/ml) in the presence or absence of Ramos pretargeted with anti-CD7-FITC or anti-CD19-FITC. (C) IHH were treated with an increasing dose of scFvFITC:sCD40L in the presence or absence of Ramos pretargeted with anti-CD7-FITC or anti-CD19-FITC. (D) iDC was treated with fresh medium or LPS (5 µg/ml) for 48h. DC maturation was determined by assessment of CD83, CD86 and HLA-DR expression. Statistical analysis was performed using two-way ANOVA (* $p < 0.05$, **$p < 0.01$, *** $p < 0.001$, n.s. not significant)

Supplementary Table 1 S3: Table of all FITC-labeled antibodies used for Fig 5H.

cell lines	Isotype mAb-FITC	antigen targeted mAb-FITC
**Fadu**	CD33	EpCAM
**LnCap**	CD20	PSMA
**MDA-MB-231**	CD20	HER2
**Z138**	HER2	RTX
**DLD-1**	CD33	EGFR
